# Cardiac self-efficacy and quality of life in patients with coronary heart disease: a cross-sectional study from Palestine

**DOI:** 10.1186/s12872-019-01281-7

**Published:** 2019-12-13

**Authors:** Aya Barham, Reem Ibraheem, Sa’ed H. Zyoud

**Affiliations:** 1 Department of Medicine, College of Medicine and Health Sciences, An-Najah National University, Nablus, 44839 Palestine; 2 Poison Control and Drug Information Center (PCDIC), College of Medicine and Health Sciences, An-Najah National University, Nablus, 44839 Palestine; 3 Department of Clinical and Community Pharmacy, College of Medicine and Health Sciences, An-Najah National University, Nablus, 44839 Palestine; 4 Clinical Research Centre, An-Najah National University Hospital, Nablus, 44839 Palestine

**Keywords:** Cardiac self-efficacy, Quality of life, Coronary heart disease, Palestine

## Abstract

**Background:**

Psychological factors, such as self-efficacy, are important in understanding the progress and management of coronary heart disease (CHD), and how patients make lifestyle modifications to compensate for the disease. The main objectives of this research are to assess patterns of cardiac self-efficacy (CSE) and quality of life (QoL) among CHD patients, and to determine the factors that affect their QoL.

**Methods:**

A cross-sectional descriptive correlational study was carried out between August 2016 and December 2016. We used a structured questionnaire completed by interviewers during face-to-face interviews with patients. Cardiac self-efficacy was evaluated using three scales: 1) the 5-item perceived efficacy in patient- physician interaction scale (PEPPI-5); 2) the self-efficacy for managing chronic diseases 6-item scale (SEMCD-6) and 3) Sullivan’s cardiac self-efficacy scale 13-items (SCSES). The 5-level version of the EuroQoL 5-dimensions questionnaire (EQ-5D-5 L), and Euroqol Visual Analogue Scale (EQ VAS) were used to evaluate health-related QoL (HRQoL) among CHD patients. Multiple binary logistic regression was carried out to evaluate the influence on the QoL score of demographic and medical characteristics, and self-efficacy factors.

**Results:**

A total of 275 patients participated in our study. The patients’ mean age was 59.51 ± 1.005 years. The HRQoL was measured by the EQ-5D-5 L index score and EQ-VAS score; their means were 0.62 ± 0.16 and 57.44 ± 1.61, respectively. The QoL showed moderate positive correlations with the PEPPI-5 (*r* = 0.419; *p*-value < 0.001), SEMCD-6 (*r* = 0.419; *p*-value < 0.001), and SCSES score (*r* = 0.273; *p*-value < 0.001). Multiple binary logistic regression showed that only patients with higher PEPPI-5 score (odds ratio (OR) = 1.11; 95% confidence interval (CI) =1.01–1.22; *p* = 0.036), and higher SCSES score (OR = 1.10; 95% CI = 1.03–1.17; *p* = 0.004) were significantly associated with a high QoL score. Moreover, multiple binary logistic regression model showed that patients with higher numbers of medications (OR = 0.23; 95% CI = 0.07–0.78); *p* = 0.018) remained significantly associated with impaired QoL.

**Conclusions:**

Lower levels of self-efficacy and poorer patient-physician interactions predicted poor HRQoL. Thus, health providers should be aware of these factors in CHD patients when trying to improve their QoL.

## Background

Coronary heart disease (CHD) results from atherosclerotic changes of the coronary artery. Hypertension, diabetes mellitus, obesity, smoking and an aggressive response to stress are well-known major risk factors for the development of CHD [1]. In most countries, CHD is considered to be one of the important causes of global morbidity and mortality and is a major economic burden. In developed countries, the mortality rate due to CHD has recently decreased; however, the morbidity rate has increased [2, 3].

In the occupied Palestinian territory, cardiovascular diseases are one of the main causes of morbidity and mortality. They are the first leading cause of death in Palestine, as reported by the Palestinian Ministry of Health in 2010, accounting for approximately 25% of all deaths, followed by cerebrovascular diseases (12%), cancer (11%) and diabetes (6%) [4]. Coronary heart disease was the cause of approximately 2.3% of all reported deaths in Palestine in 2014; most deaths due to CHD occur above the age of 65 years. Coronary heart disease is the main cause of death among other cardiovascular diseases in Palestine, accounting for about 36.1% of all cardiovascular deaths in the West Bank in 2014 [5]. A study of CHD conducted in Jerusalem in 1997, in the Palestinian and Jewish population, showed that the rate of CHD in Palestinian women was 2.4 times more than that in Jewish women, and the rate in Palestinian men was 1.6 times more than that in Jewish men [6].

In addition to cardiac physiology, psychological factors are important in understanding the progress and management of any chronic disease, and how new lifestyle modifications can compensate for the disease. Psychological factors, for example, anxiety and cardiac self-efficacy (CSE), may affect the development of chronic diseases through both psychological distress and patient behaviours. Cardiac self-efficacy is defined as a cardiac-specific measure of a patient’s confidence in his or her capacity to carry out activities which may be affected by symptoms and complications of their cardiovascular disease [7].

Because of illness manifestations following CHD, the health-related quality of life (HRQoL) could be harmed. The World Health Organization describes quality of life (QoL) as “… individual’s realisation of his/her position in life in the context of the prevailing culture and beliefs and in relation to his/her goals and concerns” [8]. In modern medicine, QoL is as a predictor of general well-being that is an important outcome in the treatment of any chronic disease. Outcomes of treatment of any chronic disease are not merely predicted by the frequency and severity of the disease, but also by how this treatment will affect the patient’s QoL and general well-being. Quality of life in CHD patients is affected by many factors such as gender, social support, personality, socioeconomic factors, psychological symptoms (e.g. depression and anxiety), angina, and dyspnoea [9].

There is no doubt that chronic diseases has an important and adverse effect on QoL, and it is well known that improvement in it is the final and an important goal of family medicine [10]. Till now, there is a lot of physicians who focus merely on the physical aspect of diseases, despite its importance, it is not the only aspect to care of; a good doctor is the one who helps the patient to achieve a better QoL, in terms of physical, psychological, mental and social life. Family medicine focuses on several ways for improvement in QoL. An important way for achieving this is by engorging patients to participate in decision making in issues that relate to their health and disease management, when the patient understands the disease and the best way to deal with it, this will capable him/ her to live with the disease and try to minimize its adverse effects on him/ her life. A holistic approach to the patient’s physical and psychosocial well-being, a focus on the family, an emphasis on QoL, and continuity of care are main principles that make the family physician exclusively appropriate to care for chronically ill patients, as patient-centered indices of quality [11, 12].

Our purposes in this research are to assess patterns of CSE and QoL among CHD patients, and to determine the factors associated with QoL. This research is necessary because: (1) it concerns CHD, a highly prevalent disease with a high mortality rate, (2) understanding the onset, treatment and progress of CHD is achieved by CSE, QoL and life style modifications, (3) in family medicine, the concept of self-efficacy has extended far beyond being merely a psychological issue to an important concept that will affect patients’ behaviours in the treatment of chronic diseases; that is, for example, how strictly he or she will adhere to the medication schedule, eat a healthy diet, and avoid a sedentary lifestyle, (4) self-efficacy is a modifiable characteristic; many behaviours and life style modifications have been shown to improve a patient’s self-efficacy. Consequently, the findings of this study will benefit society, considering that CSE plays an important role in improving QoL in CHD patients. Thus, family physicians can achieve a high health status of their patients by applying the recommended approach deduced from the results of this study, and (5) until now, no studies have been conducted in Palestine of the effect of CSE on QoL in CHD patients.

## Methods

### Study design

A cross-sectional descriptive correlational study was conducted between August 2016 and December 2016. In-patients with CHD were recruited from the Al-Watani Hospital and Al-Najah National University Hospital, both located in Nablus city in the occupied Palestinian territories. We chose to examine CHD for two reasons. Firstly, the management of CHD is a multidisciplinary issue and includes not only a commitment to taking the prescribed drugs, but also to make lifestyle changes to cope with this chronic disease. Secondly, in the setting of many chronic diseases, higher self-efficacy has been linked to better management. We aimed to detect the relationship between CSE and QoL.

### Participants and setting

The required sample size was estimated at 275 patients. We included patients who: 1) were aged ≥18 years; 2) had a history of ischaemic heart disease; 3) had a history of coronary revascularisation; 4) had no history of myocardial infarction in the previous 6 months; 5) were permanently resident in Nablus; 6) agreed to participate in the study. We excluded patients who had acute and serious conditions that would affect their QoL or make them unable to participate in completing the interview, such as stroke, uncompensated heart failure, a body mass index (BMI) > 40, psychological problems, amputated limbs or receiving chemotherapy.

### Sampling procedure and sample size calculation

According to the American Heart Association, the total CHD prevalence was 6.4% in the United States in adults older than 20 years of age [13]. In addition, a previous study by Kark et al. [6] found that the incidence of coronary events among Palestinians in Jerusalem was 0.34%. Another study in Kerala, South India found that the prevalence of CHD was 7.4% for rural Kerala and 11% for urban Kerala [14]. Another study by Suwatanaviroj and Yamwong [15] found that the prevalence of definite CHD in Thai Muslims was 14.20%, which was significantly higher than the prevalence in Thai Buddhists (6.2%). Furthermore, projections from 2013 estimates show that the prevalence of CHD will increase by nearly 18% by 2030 [16]. Thus, we took the highest percentage into consideration and, using the Raosoft sample size calculator: (http://www.raosoft.com/samplesize.html), an automated software program, the number of 225 was reached. In addition, another 5 to 10% was added in an attempt to minimise errors and increase the study’s reliability as much as possible. In the current study, 275 CHD patients were included. A convenience sample of participant was recruited.

### Data collection instrument

The data collection instruments contained four sections.

The first section gathered socio-demographic data which were provided by participants, such as age (< 48, 48–57, 58–67, 68–77, or ≥ 78), gender (male or female), residence (city, village, or Palestinian refugee camp), marital status (married, single, widowed, or divorced), educational level (no formal, primary or secondary school, or university), occupation (employed or unemployed), income (moderate to high or low), and height and weight information. We calculated the BMI of each participant using the Excel program (defined as “weight in kilograms divided by height in metres squared”) and categorised this into underweight (< 18.50), normal (18.50–24.99), overweight (≥25.00), and obese (≥30.00) [6] .

- The second section recorded clinical CHD-associated data, such as duration of the illness in years (≤3, 4–5, or ≥ 5), total number of medications (1–3, 4–6, or ≥ 7), the number of associated chronic morbid conditions (0, 1, 2, 3, or ≥ 4), and smoking (none, light, moderate, heavy, or ex-smoker).
- The third section concerned CSE, which was evaluated using three scales: 1) the 5-item perceived efficacy in patient-physician interaction scale (PEPPI-5). The PEPPI-5 includes five items; each item starts with “how confident are you in your ability to..? ” Items are rated by participants from one to five; 1 = “not at all confident”, 5 = “very confident”. The totalled results are in the range of 5 to 25; higher scores indicate that the participant has higher self-efficacy in patient-physician interactions [17]. 2) The self-efficacy for managing chronic disease 6-item scale (SEMCD-6) consists of six items, each starting with “How confident are you that you can...?” Participants rated each item on a 10-point scale; 1 = “not at all confident “, 10 = “totally confident”. Total scores of this scale are totalled to range from 6 to 60, with higher scores representing higher perceived self-efficacy for managing chronic diseases [18]. 3) Sullivan’s cardiac self-efficacy scale 13-items (SCSES); this was originally written in English and put through the standard process of translation into Arabic and back-translation into English by independent bilingual translators to assess its translational validity. Sullivan et al. developed the SCSES, which consists of 13 items, each starting with: ‘how confident are you that you know or can … ’. Items are rated by participants from 0 to 4: 0 = “not at all confident”, 4 = “completely confident”. This scale contains two dimensions: control symptoms (8 questions) and maintaining functioning (5 questions) [19].
- The last section was related to QoL: we used the 5-level version of the EuroQoL 5-dimensions questionnaire (EQ-5D-5 L), and Euroqol Visual Analogue Scale (EQ-VAS). The EQ-5D-5 L is designed to collect information related to QoL from five health domains: self-care, mobility, usual activity, anxiety/depression, and pain/discomfort. Each aspect of these domains was evaluated in terms of no/slight/moderate/severe/extreme problems. The EQ-VAS asks the participant to rate his/her overall health by marking an X on a scale from 0 to 100 [20]. The EuroQol Group offered the Arabic version of the EQ-5D by online registration (ID: 15871). The EQ-5D score was calculated using the UK value set [21] due to the lack of a Palestinian or regional value set at the time of this study; this value set was the most commonly used in Palestine [22–25]. We performed a pilot study on 15 patients to test the feasibility and clarity of the questions. The patients participating in the pilot study were not included in the final analysis. Trained medical students, under the continuous supervision of research team members, completed the data collection forms in face-to-face interviews with CHD patients. The face and content validity of the instrument was established by three experts in biostatistics as well as in research related to QoL.

### Ethics approval

Approval to conduct the study was given by the An-Najah National University Institutional Review Board (IRB). Authorisation from the Palestinian health authorities was also obtained.

### Statistical analysis

All analyses were accomplished using the Statistical Package for Social Sciences (SPSS, version 15). All data were presented as mean ± standard deviation or median [interquartile range], or frequency (percentage), as appropriate. The patients were categorised into two groups using the median utility indexes (low or high) [26–28]. For EQ-5D index value and EQ-VAS, the HRQoL was considered high, if the index was ≥ median, and low, if the index was < median. The normality of the data distribution was assessed by the Kolmogorov-Smirnov test. For the continuous variables the Mann Whitney test was used, whereas for the categorical variables, Chi-square test was used. Additionally, Spearman’s rank correlation coefficients were calculated. Multiple binary logistic regression analysis was carried out to evaluate the influence of demographic and medical characteristics, and self-efficacy factors on the QoL score. Multiple binary logistic regression analysis was used to identify factors associated with better HRQoL by including the factors, which were statistically significant in the univariate analysis. Statistical significance was set at *p* < 0.05. Internal consistency for all scales was measured using Cronbach’s alpha coefficient.

## Results

### Participant’s characteristics

A total of 275 patients agreed to participate in the current study, giving a response rate of 97.17%. The patients’ mean age was 59.51 ± 10.1 years with a range of 29 and 90 years. As seen in Table 1, most were male (56%), married (72%), had village residency (49.1%), were unemployed (53.1%), had a low income (57.8%), a primary level of education (38.5%), and were nonsmokers (52.4%). The mean duration of the disease was 4.09 ± 3.80. Thirteen (4.7%) had no history of chronic diseases, 38 (13.8%) had a history of one disease, 84 (30.5%) had a history of two diseases, 73 (26.5%) had a history of three diseases, and 67 (24.4%) had a history of four or more diseases. Eighty-one (29.5%), 161 (58.5%), and 33 (12.0%) were intending to use one to three, four to six, and more than six medications, respectively. The mean BMI was 28.78 ± 4.03.

**Table 1:** Socio-demographic and clinical characteristics of the study sample

Variable	Frequency (%) *N* = 275
Age (year)	
< 48	28(10.2)
48–57	77(28.0)
58–67	114(41.5)
68–77	43(15.6)
≥ 78	13(4.7)
Gender	
Female	121(44.0)
Male	154(56.0)
Residency	
City	108 (39.3)
Village	135(49.1)
Palestinian refugee camps	32(11.6)
Social status	
Married	198(72.0)
Single, widowed, divorced	77(28.0)
Educational level	
No Formal	66(24.0)
Primary	106(38.5)
Secondary	60(21.8)
University	43(15.6)
Occupation	
Employed	129(46.9)
Unemployed	146(53.1)
Income (NIS)	
Moderate to High	116(42.2)
Low	159(57.8)
BMI	
Normal	49(17.8)
Overweight	129(46.9)
Obese	97(35.3)
Cigarette	
Not smoker	144(52.4)
Light	7(2.5)
Moderate	36 (13.1)
Heavy	13 (4.7)
Ex-smoker	75 (27.3)
Duration of the disease (year)	
≤ 3	139 (50.5)
4–5	71 (25.8)
> 5	65 (23.6)
Total number of medications	
1–3	81(29.5)
4–6	161(58.5)
≥ 7	33(12.0)
Total number of chronic diseases	
0	13 (4.7)
1	38 (13.8)
2	84 (30.5)
3	73 (26.5)
≥ 4	67 (24.4)

*EQ-5D* European Quality of Life scale 5 dimensions, *EQ-VAS* European Quality of Life visual analogue scale, *NIS* New Israeli Shekel, *BMI* body mass index

### EQ-5D health status

**Fig. 1:**
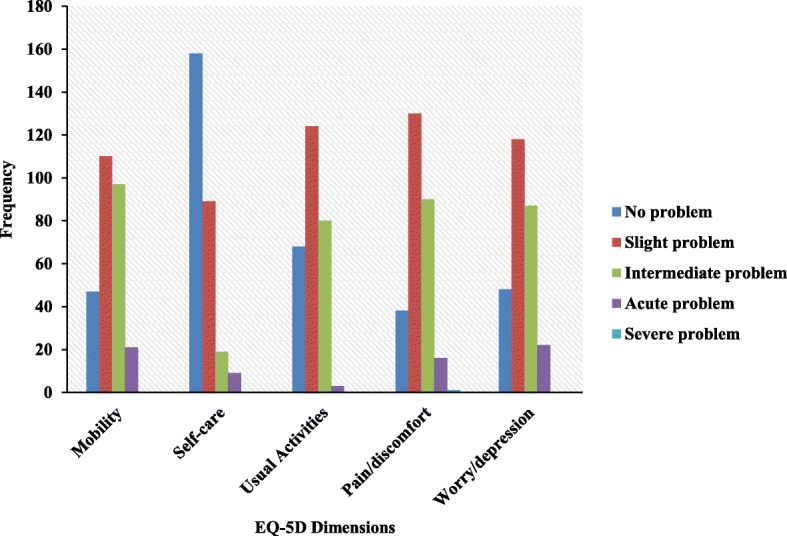
Distribution of health-related quality of life measures in different European quality of life scale 5 (EQ-5D) dimensions

The number (%) of patients who reported that there was no problem in each dimension of the five QoL dimensions was as follows: mobility 47 (17.1%), self-care 158 (57.5%), usual activities 68 (24.7%), pain/discomfort 38 (13.8%) and anxiety/depression 48 (17.5%), as shown in Fig1. A total of 97 health states were found, and 3 (1.1%) participants reported that they had no problem in any QoL dimension.

### EQ-5D index values and EQ-VAS score

The median and mean of the EQ-5D index value was 0.64 [interquartile range: 0.56–0.73], 0.62 (SD 0.16), respectively. The estimates of internal consistency (Cronbach’s alpha) for the EQ-5D index was 0.755, indicating acceptable internal consistency reliability. The median and mean of the EQ-VAS was 60.00 [interquartile range: 45.00–70.00] and 57.44 ± 1.61, respectively. The cut-off for impaired HRQoL was 0.64 and 60 for EQ-5D index value and EQ-VAS, respectively. Of the 275 CHD patients, 140 (50.9%) patients had high EQ-5D index value, and 148 (53.8%) had high EQ-VAS score. Table 2 shows the significant differences between participants according to age, occupational status, duration of disease, total number of medications, total number of chronic diseases, PEPPI-5, SEMCD-6, and SCSES (*p*-value< 0.05). No remarkable differences were noted between participants according to gender, residency, social status, educational level, income, BMI, or cigarette smoking.

**Table 2:** EQ-5D total score by socio-demographic and clinical variables

Variable	Total Frequency (%) or median [Q1-Q3] *N* = 275	Patients with better HRQoL Frequency (%) or median [Q1-Q3] *n* = 140	Patients with worse HRQoL Frequency (%) or median [Q1-Q3] *n* = 135	*p*-value ^a^
Age (year)				
< 48	28(10.2)	21(15.0)	7(5.2)	**0.025**^**b**^
48–57	77(28.0)	43(30.7)	34(25.2)	
58–67	114(41.5)	54(38.6)	60(44.4)	
68–77	43(15.6)	16(11.4)	27(20.0)	
≥ 78	13(4.7)	6(4.3)	7(5.2)	
Gender				
Female	121(44.0)	56(40.0)	65(48.1)	0.174 ^b^
Male	154(56.0)	84(60.0)	70(51.9)	
Residency				
City	108 (39.3)	54(38.6)	54(40.0)	0.820 ^b^
Village	135(49.1)	71(50.7)	64(47.4)	
Palestinian refugee camps	32(11.6)	15(10.7)	17(12.6)	
Social status				
Married	198(72.0)	103(73.6)	95(70.4)	0.554 ^b^
Single, widowed, divorced	77(28.0)	37(26.4)	40(29.6)	
Educational level				
No formal	66(24.0)	28(20.0)	38(28.1)	0.181 ^b^
Primary	106(38.5)	52(37.1)	54(40.0)	
Secondary	60(21.8)	33(23.6)	27(20.0)	
University	43(15.6)	27(19.3)	16(11.9)	
Occupation				
Employed	129(46.9)	77(55.0)	52(38.5)	**0.006**^**b**^
Unemployed	146(53.1)	63(45.0)	83(61.5)	
Income (NIS)				
Moderate to high	116(42.2)	67(47.9)	49(36.3)	0.052 ^b^
Low	159(57.8)	73(52.1)	86(63.7)	
BMI				
Normal	49(17.8)	27(19.3)	22(16.3)	0.390 ^b^
Overweight	129(46.9)	69(49.3)	60(44.4)	
Obese	97(35.3)	44(31.4)	53(39.3)	
Cigarette				
Not smoker	144(52.4)	79(56.4)	65(48.1)	0.541 ^b^
Light	7(2.5)	4(2.9)	3(2.2)	
Moderate	36(13.1)	18(12.9)	18(13.3)	
Heavy	13(4.7)	7(5.0)	6(4.4)	
Ex-smoker	75(27.3)	32(22.9)	43(31.9)	
Duration of the disease (year)				
≤ 3	139 (50.5)	82(58.6)	57(42.2)	**0.025**^b^
4–5	71 (25.8)	31(22.1)	40(29.6)	
> 5	65 (23.6)	27(19.3)	38(28.1)	
Total number of medications				
1–3	81(29.5)	25(18.5)	56(40.0)	**< 0.001**^**b**^
4–6	161(58.5)	87(64.4)	74(52.9)	
≥ 7	33(12.0)	23(17.0)	10(7.1)	
Total number of chronic diseases				
0	13(4.7)	3(2.2)	10(7.1)	**< 0.001**^**b**^
1	38(13.8)	9(6.7)	29(20.7)	
2	84(30.5)	44(32.6)	40(28.6)	
3	73(26.5)	34(25.2)	39(27.9)	
≥ 4	67(24.4)	45(33.3)	22(15.7)	
PEPPI-5	17[15–20]	18[16–20]	16[13–8]	**< 0.001**^**c**^
SEMCD-6	5.8[5–6.8]	6.3[5.4–7.3]	5.5[4.7–6.5]	**< 0.001**^**c**^
SCSES	34[29–38]	37[31–40]	31[26–36]	**< 0.001**^**c**^

*EQ-5D* European Quality of Life scale 5 dimensions, *NIS* New Israeli Shekel, *BMI* body mass index, *PEPPI-5* Perceived Efficacy in Patient-Physician Interactions, *SEMCD-6* Self-Efficacy for Managing Chronic Disease 6-Item Scale, *SCSES* Sullivan’s cardiac self-efficacy scale 13-items, *Q1-Q2* Quartile 1 - Quartile 3

^a^ The bold values indicate *P* < 0.05

^b^ Statistical significance of differences calculated using the Pearson Chi-Square test

^c^ Statistical significance of differences calculated using the Mann–Whitney U test

Table 3 shows significant differences between participants according to age, educational level, occupation, income, duration of disease, PEPPI-5, SEMCD-6, and SCSES (*p*-value < 0.05). No significant differences were noted between CHD patients according to gender, residency, social status, cigarette smoking, BMI, total number of medications, or total number of chronic diseases. A modest positive correlation was found between EQ-VAS and EQ-5D (*r* = 0.378, *p*-value< 0.001).

**Table 3:** EQ-VAS by socio-demographic and clinical characteristics

Variable	Total Frequency (%) or median [Q1-Q3] *N* = 275	Patients with better HRQoL Frequency (%) or median [Q1-Q3] *n* = 148	Patients with worse HRQoL Frequency (%) or median [Q1-Q3] *n* = 127	*p*-value ^a^
Age (year)				
< 48	28(10.2)	23 (15.5)	5 (3.9)	**0.008**^**b**^
48–57	77(28.0)	42 (28.4)	35 (27.6)	
58–67	114(41.5)	60 (40.5)	54 (42.5)	
68–77	43(15.6)	16 (10.8)	27 (21.3)	
≥ 78	13(4.7)	7 (4.7)	6 (4.7)	
Gender				
Female	121(44.0)	62 (41.9)	59 (46.5)	0.447 ^b^
Male	154(56.0)	86 (58.1)	68 (53.5)	
Residency				
City	108 (39.3)	57 (38.5)	51 (40.2)	0.819 ^b^
Village	135(49.1)	75 (50.7)	60 (47.2)	
Palestinian refugee camps	32(11.6)	16 (10.8)	16 (12.6)	
Social status				
Married	198(72.0)	113 (76.4)	85 (66.9)	
Single, widowed, divorced	77(28.0)	35 (23.6)	42 (33.1)	0.083 ^b^
Educational level				
No formal	66(24.0)	27 (18.2)	39 (30.7)	
Primary	106(38.5)	51 (34.5)	55 (43.3)	**< 0.001**^b^
Secondary	60(21.8)	34 (30.0)	26 (20.5)	
University	43(15.6)	36 (24.3)	7 (5.5)	
Occupation				
Employed	129(46.9)	88 (59.5)	41 (32.3)	**< 0.001**^**b**^
Unemployed	146(53.1)	60 (40.5)	86 (67.7)	
Income (NIS)				
Moderate to high	116(42.2)	79 (53.4)	37 (29.1)	**< 0.001**^**b**^
Low	159(57.8)	69 (46.6)	90 (70.9)	
BMI				
Normal	49(17.8)	24 (16.2)	25 (19.7)	0.570 ^b^
Overweight	129(46.9)	68 (45.9)	61 (48.0)	
Obese	97(35.3)	56 (37.8)	41 (32.3)	
Cigarette				
Not smoker	144(52.4)	85 (57.4)	59 (60.5)	0.257 ^b^
Light	7(2.5)	4 (2.7)	3 (2.4)	
Moderate	36(13.1)	17 (11.5)	19 (15.0)	
Heavy	13(4.7)	4 (2.7)	9 (7.1)	
Ex-smoker	75(27.3)	38 (25.7)	37 (29.1)	
Duration of the disease (year)				
≤ 3	139 (50.5)	86(58.1)	53(41.7)	
4–5	71 (25.8)	38(25.7)	33(26/0)	**< 0.001**^b^
> 5	65 (23.6)	24(16.2)	41(32.3)	
Total number of medications				
1–3	81(29.5)	47 (31.8)	34 (26.8)	0.055 ^b^
4–6	161(58.5)	78 (52.7)	83 (65.4)	
≥ 7	33(12.0)	23 (15.5)	10 (7.9)	
Total number of chronic diseases				
0	13(4.7)	11 (7.4)	2 (1.6)	
1	38(13.8)	22 (14.9)	16 (12.6)	0.070 ^b^
2	84(30.5)	37 (25.0)	47 (37.0)	
3	73(26.5)	41 (27.7)	32 (25.2)	
≥ 4	67(24.4)	37 (25.0)	30 (23.6)	
PEPPI-5	17[15–20]	18[16–20]	16[14–19]	**0.002**^c^
SEMCD-6	5.8[5–6.8]	6.1[5.3–6.8]	5.5[4.8–6.8]	**0.005**^c^
SCSES	34[29–38]	37[30–40]	31[27–36]	**< 0.001**^c^

*EQ-VAS* European Quality of Life visual analogue scale, *NIS* New Israeli Shekel, *BMI* body mass index, *PEPPI-5* Perceived Efficacy in Patient-Physician Interactions, *SEMCD-6* Self-Efficacy for Managing Chronic Disease 6-Item Scale, *SCSES* Sullivan’s cardiac self-efficacy scale 13-items, *Q1-Q2* Quartile 1 - Quartile 3

^a^ The bold values indicate *P* < 0.05

^b^ Statistical significance of differences calculated using the Pearson Chi-Square test

^c^ Statistical significance of differences calculated using the Mann–Whitney U test

### Self-efficacy scales

The median of the PEPPI-5, SCSES and SEMCD-6 was 17.00 [interquartile range: 15.00–20.00], 34.00 [interquartile range: 29.00–38.00], and 5.80 [interquartile range: 5.00–6.80], respectively. The estimates of internal consistency (Cronbach’s alpha) for the PEPPI-5, SCSES and SEMCD-6 were 0.799, 0.848, and 0.865, respectively, indicating acceptable to good internal consistency reliability.

### Univariate analysis

The univariate analysis showed that age (*p* = 0.025), occupational status (*p* = 0.006), duration of disease (*p* = 0.025), total number of medications (*p* < 0.001), total number of chronic diseases (*p* < 0.001), PEPPI-5 (*p* < 0.001), SEMCD-6 (*p* < 0.001), and SCSES (*p* < 0.001) were significantly associated with better QoL (Table 2). Correlation tests revealed moderate positive associations between the QoL and the PEPPI-5 (*r* = 0.419; *p*-value < 0.001), SEMCD-6 (*r* = 0.419; *p*-value < 0.001), and SCSES scores (*r* = 0.273; *p*-value < 0.001); (Table 4).

**Table 4:** Correlations with quality of life in coronary heart disease patients

Scales	EQ-5D		EQ-VAS	
	*P*-value	Correlation	*P*-value	Correlation
PEPPI-5	< 0.001	0.419	< 0.001	0.419
Sullivan’s scale	< 0.001	0.524	< 0.001	0.524
SEMCD-6	< 0.001	0.273	< 0.001	0.273
EQ-5D			< 0.001	0.378
EQ-VAS	< 0.001	0.378		

*EQ-5D* European Quality of Life scale 5 dimensions, *EQ-VAS* European Quality of Life visual analogue scale, *PEPPI-5* Perceived Efficacy in Patient-Physician Interactions, *SEM-CD* Self-Efficacy for Managing Chronic Disease 6-Item Scale

### Multiple logistic regression analysis

Multiple binary logistic regression analysis, using the EQ-5D-5 L index score (high versus low) as a dependent variable and the following factors as independent variables: covariates of age, employment status, duration of disease, number of medications, number of chronic diseases, PEPPI-5, SEMCD-6, and SCSES scores, showed that only patients with higher PEPPI-5 score (odds ratio (OR) = 1.11; 95% confidence interval (CI) = 1.01–1.22; *p* = 0.036), and higher SCSES score (OR = 1.10; 95% CI = 1.03–1.17; *p* = 0.004) were significantly associated with a high QoL score. Moreover, multiple binary logistic regression model showed that patients with higher numbers of medications (OR = 0.23; 95% CI = 0.07–0.78); *p* = 0.018) remained significantly associated with impaired QoL. The results of the multiple binary logistic regression model are summarised in Table 5.

**Table 5:** Patients characteristics associated with quality of life in multiple binary logistic regression model

Variable		B	S.E.	Wald	Sig.	Odds ratio with 95% CI
Age	< 48					Ref.
	48–57	−0.07	0.59	0.01	0.912	0.94(0.29–2.98)
	58–67	−0.10	0.60	0.03	0.875	0.91(0.28–2.95)
	68–77	−0.08	0.71	0.01	0.910	0.92(0.23–3.70)
	≥78	1.64	0.99	2.75	0.097	5.16(0.74–35.93)
Occupation	Unemployed					Ref.
	Employed	0.27	0.32	0.74	0.391	1.31(0.71–2.43)
Duration of disease (year)	≤3					Ref.
	4–5	−0.78	0.37	4.33	0.057	0.46(0.22–1.09)
	> 5	−0.69	0.42	2.71	0.100	0.50(0.22–1.14)
Total number of medications	1–3					Ref.
	4–6	−0.63	0.40	2.48	0.115	0.54(0.25–1.17)
	≥ 7	−1.47	0.62	5.57	**0.018**	0.23(0.07–0.78)
Total number of chronic diseases	0					Ref.
	1	0.18	0.96	0.04	0.853	1.19(0.18–7.77)
	2	−0.91	0.91	1.00	0.317	0.40(0.07–2.39)
	3	−0.37	0.94	0.16	0.691	0.69(0.11–4.33)
	≥4	−1.10	0.97	1.30	0.254	0.33(0.05–2.21)
Patient-Physician Interactions score		0.10	0.05	4.41	**0.036**	1.11(1.01–1.22)
Self-Efficacy for Managing Chronic Disease score		0.26	0.14	3.34	0.068	1.29(0.98–1.70)
Cardiac Self-Efficacy score		0.09	0.03	8.51	**0.004**	1.10(1.03–1.17)

*CI* confidence interval, *β* coefficient of predictor variables, *S.E* standard error

^a^ The bold values indicate *P* < 0.05

## Discussion

The main objective of this research was to assess patterns of CSE and QoL in CHD patients in Nablus, Palestine. The EQ-5D and EQ-VAS scales were used to evaluate QoL. The SEMCD-6, SCSES and PEPPI-5 were used to assess CSE.

In our study, the mean EQ-5D score among the CHD patients was 0.62 ± 0.16, whereas the findings in Chinese, Slovenian and Swiss studies using the same instrument were 0.889 ± 0.172 [29], 0.60 ± 0.19 [30], 0.82 ± 0.16 [31], respectively. However, studies of hypertensive, end stage renal disease and diabetic patients in our country revealed the means of the EQ-5D were 0.80 ± 0.16 [22], 0.44 ± 0.37 [24], 0.7 ± 0.20 [32], respectively.

Using univariate analysis, independent associations with high QoL scores tended to be found in employment patients; in patients with short duration of the disease; a low number of medications; a low number of co-morbidities; and high score in the PEPPI-5, SEMCD-6, and SCSES instruments. Multiple binary logistic regression model showed that only patients with lower numbers of medications, and higher PEPPI-5 and SCSES scores, were associated with high QoL. Our findings seem to be consistent with those of other studies that found an association between self-efficacy and QoL [7, 33–35]. There are several patient behaviours that may affect the progress of chronic diseases, such as adherence to a healthy diet, medications and healthy lifestyle, and avoiding unhealthy activities such as smoking and a sedentary lifestyle [2, 35]. Numerous studies have shown that psychological distress is one of the major risk factors that may contribute to the development of CHD, in addition to the well-known risk factors such as hypertension and obesity [2]. Furthermore, type 2 diabetes may act as a negative factor that results in a poor quality of life, worse prognosis and severe clinical complications in patients with stable and unstable CHD [36–40].

It is proposed that health outcomes of the treatment of any chronic disease are affected by the patient’s beliefs in his or her capability to adhere to medications and follow a healthy lifestyle. It is this belief which actually affects their behaviours [35]; according to Bandura, if people lack the self-efficacy to do something, they will not do it in the best way even if they can do it very well [41].

A modest positive correlation between EQ-5D and EQ-VAS was found with means for each as follows: 0.62 ± 0.16 and 57.44 ± 1.61, similar to those reported in a Slovenian study in which the EQ-5D and EQ-VAS were 0.60 ± 0.19 and 58.6 ± 19.9, respectively [30]. These findings are in line with those of previous Palestinian studies with different populations, such as those with diabetes [32], hypertension [25], or chronic kidney disease [24].

Our results found that young patient age was associated with a high QoL, which accords with previous studies [29, 42]. This can be explained by the observation that older patients have a longer duration of the disease, more co-morbidity and are more vulnerable to fear and a sense of approaching death. In addition, unemployment was correlated with a low QoL, as demonstrated by previous studies [43, 44]; possible explanations are that occupation raises access to health care by providing health insurance, assures income, and increases patient self-confidence.

The domain of clinical factors (duration of the disease, number of co-morbidities and number of medications) had an impact on QoL. A long duration of the disease, and high numbers of co-morbidities and medications were associated with low QoL, as found in other studies [45].

This study’s strengths lie in the following: (1) it is the first study conducted in Palestine to assess the effects of CSE on QoL in CHD patients, (2) the sample size is relatively large, making it possible to identify the different factors that affect CSE and QoL and the relationship between them in CHD patients; (3) we conducted our research on CHD patients who were referred to Al-Watani Hospital and Al-Najah National University Hospital; most CHD patients in Nablus city, and the villages and refugee camps around it, receive their inpatient care in these hospitals. Consequently, the results of this research can be generalised to the Nablus population; and (4) data collection was cost-efficient. However, we faced some limitations during the conduct of this research, as follows: (1) this is a cross-sectional study and it is therefore difficult to prove causal relationships between the scales and their associated factors; and (2) the results of this research cannot be generalised to the overall Palestinian population since data were collected only from the Nablus population.

## Conclusions

The results of the study identified that lower levels of self-efficacy, poorer patient-physician interactions, and patients with high number of medications predicted lower HRQoL. Health providers should be aware of these factors in CHD patients when attempting to improve their QoL.

## Data Availability

The datasets used and/or analysed during the current study are available from the corresponding author on reasonable request.

## Abbreviations

BMI: Body mass index
CHD: Coronary artery disease
CI: Confidence interval
CSE: Cardiac self-efficacy
EQ-5D-5 L: Five-level EuroQoL five-dimensional instrument
HRQoL: Health-related quality of life
IRB: Institutional review board
OR: Odds ratios
PEPPI-5: 5-item perceived efficacy in patient-physician interaction scale
Q1-Q2: Quartile 1 - quartile 3
QoL: Quality of life
SCSES: Sullivan’s cardiac self-efficacy scale
SD: Standard deviation
SEMCD-6: Self- efficacy for managing chronic diseases 6-item scale
